# Natural Biostimulant Attenuates Salinity Stress Effects in Chili Pepper by Remodeling Antioxidant, Ion, and Phytohormone Balances, and Augments Gene Expression

**DOI:** 10.3390/plants10112316

**Published:** 2021-10-27

**Authors:** Alaa I. B. Abou-Sreea, Clara R. Azzam, Sudad K. Al-Taweel, Ranya M. Abdel-Aziz, Hussein E. E. Belal, Mostafa M. Rady, Atef A. S. Abdel-Kader, Ali Majrashi, Khaled A. M. Khaled

**Affiliations:** 1 Department of Horticulture, Faculty of Agriculture, Fayoum University, Fayoum 63513, Egypt; aib00@fayoum.edu.eg; 2 Cell Research Department, Field Crops Research Institute, Agricultural Research Center, Giza 12619, Egypt; 3 Department of Field Crops, College of Agriculture Engineering Sciences, University of Baghdad, Al-Jadiriya, Baghdad 10070, Iraq; sudad.altaweel@coagri.uobaghdad.edu.iq; 4 Tissue Culture Lab., Sugar Crops Research Institute, Agriculture Research Center, Giza 12619, Egypt; snowrosa114@gmail.com; 5 Botany Department, Faculty of Agriculture, Fayoum University, Fayoum 63514, Egypt; hes00@fayoum.edu.eg (H.E.E.B.); mmr02@fayoum.edu.eg (M.M.R.); 6 Department of Medicinal and Aromatic Plants, Horticulture Research Institute, Agricultural Research Center, Giza 12619, Egypt; atef_om@yahoo.com; 7 Department of Biology, College of Science, Taif University, P.O. Box 11099, Taif 21944, Saudi Arabia; aa.majrashi@tu.edu.sa; 8 Genetics Department, Faculty of Agriculture, Beni-Suef University, Beni Suef 62513, Egypt; khaled.adly@agr.bsu.edu.eg

**Keywords:** *Capsicum frutescens* L., natural honey bee, silymarin, salt stress, SCoT marker

## Abstract

A biostimulant is any microorganism or substance used to enhance the efficiency of nutrition, tolerance to abiotic stress and/or quality traits of crops, depending on its contents from nutrients. Plant biostimulants like honey bee (HB) and silymarin (Sm) are a strategic trend for managing stressed crops by promoting nutritional and hormonal balance, regulating osmotic protectors, antioxidants, and genetic potential, reflecting plant growth and productivity. We applied diluted honey bee (HB) and silymarin-enriched honey bee (HB- Sm) as foliar nourishment to investigate their improving influences on growth, yield, nutritional and hormonal balance, various osmoprotectant levels, different components of antioxidant system, and genetic potential of chili pepper plants grown under NaCl-salinity stress (10 dS m^‒1^). HB significantly promoted the examined attributes and HB-Sm conferred optimal values, including growth, productivity, K^+^/Na^+^ ratio, capsaicin, and Sm contents. The antioxidative defense components were significantly better than those obtained with HB alone. Conversely, levels of oxidative stress markers (superoxide ions and hydrogen peroxide) and parameters related to membrane damage (malondialdehyde level, stability index, ionic leakage, Na^+^, and Cl^−^ contents) were significantly reduced. HB-Sm significantly affects inactive gene expression, as a natural biostimulator silencing active gene expression. SCoT primers were used as proof in salt-treated or untreated chili pepper plants. There were 41 cDNA amplicons selected by SCoT-primers. Twenty of them were EcDNA amplicons (cDNA-amplicons that enhanced their genes by one or more treatments) representing 49% of all cDNA amplicons, whereas 7 amplicons for ScDNA (whose genes were silenced in one or more treatments) represented 17%, and 14 McDNA (monomorphic cDNA-amplicons with control) amplicons were represented by 34% from all cDNA amplicons. This indicates the high effect of BH-Sm treatments in expression enhancement of some inactive genes and their silenced effect for expression of some active genes, also confirming that cDNA-SCoT markers succeeded in detection of variable gene expression patterns between the untreated and treated plants. In conclusion, HB-Sm as a natural multi-biostimulator can attenuate salt stress effects in chili pepper plants by remodeling the antioxidant defense system and ameliorating plant productivity.

## 1. Introduction

Peppers (*Capsicum* spp.), including chili pepper (*Capsicum frutescens* L.) of the *Solanaceae* family that grow worldwide, have the most significant and common medicinal properties. It is mainly cultivated in warm climates, including in Egypt. It is vital for vitamins (e.g., A, C, and E) and secondary products, including flavonoids, phenolic acids, and carotenoids [1]. The pepper fruit has high economic significance and nutritional value, making it a brilliant source of natural color and high antioxidants for human health, also denoting it as food or functional ingredients. Thus, the spread of chili pepper should be encouraged to obtain more antioxidants related to human health [2,3,4].

Chili peppers are susceptible or moderately susceptible to salinity, which poisons plant cells due to the formation of reactive oxygen species (ROS) [5]. Plant metabolism is discouraged by salinity-induced ROS due to osmotic stress, Na^+^, and Cl^−^ ions toxicity, which contribute to the inhibition of plant growth, various physio-chemical attributes, and outputs [6,7,8,9,10]. To overcome osmotic and ionic stress, plants alter their biochemical mechanisms and stimulate defense mechanisms by synthesizing more compounds ranging from low-molecular-mass antioxidants to compatible solutes [11,12]. As a score of salinity stress, oxidative stress markers, namely, ROS (e.g., O_2_^•−^, ^1^O_2_, H_2_O_2_, and OH^−^), are created, causing extreme damage in plants [13,14,15]. Plants increase ROS-eliminating enzymes like superoxide dismutase (SOD), peroxidase (POD), catalase (CAT), ascorbate peroxidase (APX), glutathione reductase (GR), etc., as defense mechanisms under salt stress [16,17]. Collectively, there are abundant salinity-resistant mechanisms related to plant physiology and molecular biology. To support the plant to defend against the stress influences and enhance its efficiency in this regard, external aids such as eco-friendly biostimulants and antioxidants are used [18,19].

Biostimulants have been used to enhance plants’ tolerance to numerous stresses, including salinity [18,19,20,21,22,23,24]. All previously used biostimulants, including honey bee (HB), led to an increase in plant antioxidant defense systems (e.g., enzymes, low molecular weight antioxidants, and osmolytes) and promoted all components related to plant morphology, biochemistry including ionic and hormonal balances, and molecular biology [13,19,22,23,24]. The promoting mechanisms of biostimulants for stressed plants are not adequately known because of the heterogeneous nature of the different components of the biostimulator. Moreover, the reinforcing influences of a biostimulant are often the consequence of the synergistic action of two or more components together in various manners. Lately, biostimulants have been used exceedingly because of their high antioxidant capacity to eliminate or attenuate the harmful influences of stressors, including salinity. It rebalances nutrient ions and phytohormones and reinforces plants’ tolerance to stress to restore plant development, biochemistry, physiology, and molecular biology [18,19,20,21,22,23].

Honey bee (HB) is one of the recently used biostimulants to support stressed plants due to its high antioxidant activity and inorganic ions (e.g., K, P, Mg, Ca, S, Fe, Mn, Zn, Cu, I, Na, Se, etc.), various soluble sugars, organic acids, proteins, amino acids, proline, antioxidants, vitamins, and lipids [23,25]. HB enhances the plants’ salt tolerance by rebalancing nutrients, osmoprotectants, hormones, and non-enzymatic and enzymatic antioxidants, consequently strengthening the plants’ antioxidant defenses [23].

Silymarin (Sm) is extracted from the *Silybum marianum* plant as an essential secondary metabolite among the antioxidants recently used to support stressed plants individually or as an additive to enrich a biostimulant [18]. It combines six flavonolignans ingredients: silybin A and B, isosilybin A and B, silychristin, and silydianin, along with taxifolin (flavonoid). It can promote plant productivity because of its accumulation in stressed plants as a potent antioxidant to reinforce their defensive systems [26]. As reported in a unique report [18], Sm was used alone (at 0.5 mM) or to enrich a biostimulant (at 0.24 g L^−1^); both treatments were successful in suppressing oxidative stress, raising plant defense systems and antioxidant gene expressions, and minimizing cadmium toxicity in maize plants. There is a need for comprehensive research to explore the HB and Sm mechanisms of plant tolerance to stress in this interest.

Molecular markers are used to identify genotypes and determine target gene expression related to abiotic stress [27,28,29,30,31,32]. Genetic markers, such as amplified fragment length (AFLP) and start codon targeted marker (SCoT), are used to identify the gene expression in plants [33,34]. The technique based on cDNA-SCoT is an appropriate potent tool for identifying gene expression variations, gene stability, and stress tolerance [33]. This technique has an advantage over other methods because it is more efficient, simpler to operate, fast results easily replicated, and less expensive [35]. cDNA-SCoT is successfully used to determine gene expressions in *Saccharum officinarum*, *Phoenix dactylifera*, *Dendrobium Officinale*, *Mangifera indica*, and olive trees [11,36,37].

Most studies have tested the influence of biostimulants in short-term experiments on plant growth and productivity. Still, no investigations have been conducted using diluted honey bee enriched with silymarin (HB-Sm) as a multi-biostimulant to overcome the effects of salt stress on chili pepper plants. This research is the first investigation in which HB-Sm was sprayed as foliar nourishment to encourage the growth and production of chili pepper plants grown under NaCl (10 dS m^‒1^) stress. Therefore, this work aimed at examining the influence of foliar spraying of chili pepper plants with HB or HB-Sm as a multi-biostimulant to attenuate the harmful effects of salinity stress on plant growth production, physio-biochemical constituents, antioxidant defense system components, and some parameters related to metabolism. This study hypothesizes that treating the salt-stressed chili pepper plants with HB-Sm will outdo HB in raising plant salt tolerance by increasing plant metabolism and antioxidant defense system components.

## 2. Materials and Methods

### 2.1. Trial Location and Climatic Conditions

A pot trial was conducted using three on-farm greenhouses (29°17′ N; 30°53′ E, 24 m above sea level) at the College of Agriculture, Fayoum University, Fayoum Province, Egypt. The trial was implemented three times simultaneously in the 2020 summer season. Throughout the experimental period from 28 February to 1 June, average climatic conditions were 33 ± 5/19 ± 2 °C for average day/night temperatures, 66 ± 3% for average relative humidity, and 13 h for average daylight length. As for light intensity, natural sunlight was appropriate for all stages of chili pepper growth.

### 2.2. Plant Material, Experimental Layout, and Cultural Practices

Chili pepper seeds were provided by the Agricultural Research Center (ARC), Giza, Egypt. Seeds were sterilized with a 1.2% sodium hypochlorite solution for ½ h, rinsed with tap water for another ½ h, and then dried at room temperature overnight. After filling with a growth medium, plastic pots (diameter of 20 cm and depth of 18 cm) were prepared for sowing three seeds each. The medium was composed of peat moss, vermiculite, and crushed maize grains in a ratio of 1.5:1.0:0.5 (by weight), respectively, and supplemented with 250 mg humic acid L^−1^ [38]. It was mixed with a fertilizer consisting of 415 mg ammonium nitrate L^−1^, 500 mg calcium superphosphate L^−1^, 333 mg potassium sulfate L^−1^, 833 mg magnesium sulfate L^−1^, 333 mg iron L^−1^, 333 mg zinc L^−1^, 333 mg manganese L^−1^, and 1250 mg calcium carbonate L^−1^ (to adjust the pH of peat-containing media). The medium was supplemented with 125 mg wettable powder fungicide Moncut SC [25% (*w*/*w*) containing active flutolanil, Central Glass Co., Ltd., Tokyo, Japan] per L to control the medium-borne pathogens.

Three experiments were carried out at the same time in three different wire greenhouses. Each experiment included six treatments as follows: control and NaCl salt stress control (S) treated with distilled water (dH_2_O), foliar spraying with honey bee (HB) at 15 g L^−1^ or HB at 10 g L^−1^ supplemented with 0.5 mM silymarin (HB-Sm) under both salt-free and salt conditions. Selected levels of HB and Sm were identified based on results from a preliminary study (Table S1). The sprays were performed three times; at 15, 25, and 35 DAT for the stressed and unstressed plants till run-off using a hand atomizer. Each plant was sprayed with 25, 30, and 40 mL of spraying solution in the three spraying times, respectively. Each of the six treatments was accomplished with twenty pots arranged in a completely randomized design (CRD) with four replicates.

In the 25-day stage (transplants with 2–3 pairs of true expanded leaves), the transplants were uprooted and transplanted into other pots (diameter of 30 cm and depth of 32 cm), each transplant per pot, using the previous medium after supplementation with a mixture of farmyard manure and compost (5:1) at 2% and mixed with the same fertilizer mixture detailed above, containing the fungicide Moncut SC. The cultivated pots were kept in a greenhouse under normal environmental conditions described above. Ten days after transplanting (DAT), all pots were divided into two sets. In the first set, the control plants (without NaCl salt treatments) were maintained with water at regular intervals (2 days) according to the soil field capacity. Salt stress was induced in the second set by adding NaCl salt in irrigation water at 6.4 g L^−1^ (6400 ppm, 10 dS m^−^^1^), supplied day by day (from a preliminary study; Table S1). The treatments of salinity were initiated ten days after transplanting when the roots were reformed. In the saline treatments, the salinity level was controlled and kept in the growth medium at the stated concentration (10 dS m^−^^1^) by assessing, periodically, with an inductively coupled plasma atomic emission spectrometry (ICP- AES, IRIS-Advan type, Thermo, USA).

Five pots were randomly collected 50 DAT and identified for growth parameters, and another group of 5 pots was randomly collected simultaneously to estimate all physio-biochemical attributes. The ten remaining pots were collected at harvest (95 DAT) and identified to evaluate yield parameters.

### 2.3. Preparation and Analysis of Bee-Honey (HB)

Fifteen gram pure honey (when using HB alone) or 10 g pure honey [when using HB supplemented with silymarin (Sm)] was well dissolved in 1 L of dH_2_O to prepare the HB solutions. The applied HB in the trials was analyzed for its bioactive components, and the resulting data are displayed in Table 1.

### 2.4. Evaluation of Plant Parameters for Growth and Yield

Each of the five randomly selected plants was separated into the root system and shoot for assessing fresh weights, and after oven-drying at 70 °C, the root and shoot dry weights were documented after at least two constant weights. Ninety-five DAT (at harvest), ten plants were identified for assessing yield parameters; the fruits were harvested for their average number, fresh and dry weights for each plant, and capsaicin content determination.

### 2.5. Evaluation of Photosynthetic Efficiency

The instantaneous efficiency of carboxylation (iEC; µmol m^‒^^2^ s^‒^^1^), photochemical activity (via potassium cyanide technique), and photosynthetic pigment contents (mg g^‒^^1^ FW) were assessed following the procedures detailed by Konrad et al. [44]; Wellburn [45]; Avron [46], respectively, via the first fully expanding upper leaf on each plant.

The fluorescence measurements of chlorophyll a were done via a modulated fluorometer (PAM-2000, Heinz-Walz). The saturation-pulse method [44] was practiced to pre-adapted leaves to dark (12 h at 28 °C and 70% relative humidity in the growth chamber). Photosystem II quantum efficiency (Fv/Fm) was measured. Then, the plants were kept at (2 h, 28 °C, 70% relative humidity, 350 µmol m^−2^ s^−1^ of flow density of energetic photons photosynthetically (DFFFA) in the growth chamber). After measuring the net rate of CO_2_ assimilation and internal CO_2_ concentration, the iEC was computed [44]:

iEC = assimilation rate/internal concentration of CO_2_ (1)

Dimethyl sulfoxide was used to extract total chlorophyll (T. Chls) and total carotenoids (T. Carot), and the absorbances were observed at 480, 649, and 665 nm [45]. The photochemical activity (PhA) was measured by plant chloroplast fragments using the potentiometric method [46]. Capsaicin content determined as described by Nagy et al. [47].

### 2.6. Leafy Tissue Integrity Assessment

The integrity of chili pepper plant leafy tissue was examined by leaf relative water content (RWC) [48], osmolyte solutes that enable plant tissues to retain water under salt stress like free proline [49], total soluble sugars [50], choline [51], and glycine betaine [52].

The integrity of leaf tissue refers to its content of K^+^, Na^+^, and Cl^−^. After acidic digestion of dried powdered samples, contents of K^+^ and Na^+^ were measured on a Flame Photometer (Model 52-A, Perkin-Elmer) [53]. The content of chloride ion (Cl^−^) was measured on Atomic Absorption (Perkin-Elmer, Spectrophotometer) [54].

Evaluation of oxidative stress markers and membrane damages related to oxidative stress shows whether leaf tissues are in an integrity state. To quantify levels of superoxide ions (O_2_^•−^), hydrogen peroxide (H_2_O_2_), and malondialdehyde (MDA), respectively, the methods depicted by Kubis [55], Velikova et al. [56], and Heath and Packer [57] were applied. O_2_^•−^ content was assessed in leaf sample (100 mg) cut into 1 mm × 1 mm fragments and immersed for 1 h at room temperature in 10 mM K-phosphate buffer, pH 7.8, 0.05% NBT, and 10 mM NaN_3_. Two ml of the immersed solution was heated at 85 °C for 15 min and cooled rapidly. Optical density was measured calorimetrically at 580 nm, and the content was expressed as A_580_ g^−1^ FW. H_2_O_2_ content (μmol g^−1^ FW) was assessed in 250 mg of fresh leaves homogenized using 5 mL of 5% TCA (trichloroacetic acid). Centrifugation was done at 12,000× *g* for 15 min at 4 °C for the homogenates. The supernatant was gathered, added to 10 mM potassium phosphate buffer (pH 7.0) + 1 M KI as a reaction medium. The absorbance was read, spectrophotometrically, at 390 nm against H_2_O_2_ as a standard. Lipid peroxidation level was assessed as MDA content. Leaf tissue (0.1 g) was homogenized with 5 mL 0.07% NaH_2_PO_4_·2H_2_O and 1.6% Na_2_HPO_4_·12H_2_O (50 mM) and centrifuged at 20,000× *g* for 25 min. The results of MDA were expressed as A_532–600_ g^−1^ FW.

Following the procedures illustrated by Rady [58], stability index (SI) and ionic leakage (IL) were assessed for the detection of cellular membrane integrity under normal and stress conditions. These methods rely on the differences between measurement of electrical conductivity of the tissue solution before and after heating. The resulting data were computed from the following formulae:

SI (%) = [1 − (EC_1_/EC_2_)] × 100 & IL (%) = [(EC_2_ − EC_1_)/EC_3_] × 100 (2)

### 2.7. Evaluation of Non-Enzymatic Antioxidant Contents

Uniform fresh leaves on randomly chosen plants were used to quantify levels of glutathione (GSH) and ascorbate (AsA) (µmol g^‒1^ FW) [59,60], respectively. To estimate GSH, leaf samples (0.5 g) were homogenized in 2 mL of 2% solution (*v*/*v*) of metaphosphoric acid and centrifuged for 10 min at 17,000× *g*. For neutralizing the supernatant, 0.6 mL of 10% solution (*w*/*v*) of sodium citrate was added. A 1.0 mL assay was prepared by adding 100 μL of each extract, distilled H_2_O, 6 mM 5,5′-dithio-bis-2-nitrobenzoic acid and 700 μL of 0.3 mM NADPH and stabilized for 3–4 min at 25 °C. This whole assay was then added with 10 μL 5 mg mL glutathione (GSH) reductase to measure the absorbance at 412 nm. Standard curves were developed, and readings were subjected to calculating the GSH concentrations expressed on a fresh weight basis (μmol g^−1^ leaf fresh weight). To estimate the AsA content (μmol g^−1^ leaf fresh weight), the leaf extract was added to a mixture containing 30 mM of buffer (potassium phosphate, pH 7.4), TCA (2.5%), phosphoric acid (8.4%), bipyridyl (0.8%), and ferric chloride (0.3%). The reaction was conducted (40 °C, 30 min), and absorbance was recorded at 525 nm.

The procedures of Nagy et al. [47], Konings et al. [61], Ching and Mohamed [62] were followed to quantify the level of α-tocopherol (ToC) (μmol g^−1^ DW). ToC was assessed by dissolving 20 mg of HBT (butylated hydroxytoluene) using 900 mL of extraction solvent; n-hexane-ethyl acetate, n-hexane + 100 mL of ethyl acetate as a solvent mixture. Using R-Toc, standard solutions (20–200 μg mL^−1^) were prepared using a stock solution (50 mg/100 mL n-hexane). Samples were prepared and saponified [61]. After slicing, leaf tissue was dried at 40 °C with an oven, homogenized, and suspended in water by a 1 L conical flask with the addition of 21 g of KOH dissolved in 100 mL of ethanol. 0.25 g weight of AsA (ascorbic acid) was added (gram test portion). Saponification was practiced for 40 min at 80 °C, and cooling was done directly. The ethanol: water ratio was adjusted to 0.3 via distilled H_2_O, and 9 mL of n-hexane: 1 mL of ethyl acetate (3 × 100 mL) was added, and three times extraction was done for the mixtures. Then, the combination was washed with water, and the organic phases were filtered through anhydrous Na_2_SO_4_ into a beaker. Evaporation to dryness was done for the filtrates. Residues were dissolved by n-hexane (HPLC grade) and then stored at −20 °C. Toc was assessed via methanol: water (94:6) as a mobile phase (with a flow rate of 1.5 mL min^−1^, a UV detector set at 292 nm) by HPLC system (with a Waters Bondapak C18 reverse-phase column).

Total phenolic compound contents (mg kg^−1^ of tannic acid equivalent relying on the curve of calibration) were evaluated via the technique of Folin–Ciocalteu [63] with minor modification [64]. The reagent, Folin–Ciocalteu, was added to extracts using sodium carbonate. After vortexing for 15 s, color was allowed to develop to note absorbance readings of the triturate at 755 nm.

Content of silymarin (Sm) was assessed following Arampatzis et al. [65,66]. Uniform leaves randomly chosen from plants were used to extract Sm via Soxhlet apparatus. 200 mL Methanol was used for extraction. Then the extract was dried via evaporation. Subsequently, the sample was reconstituted in HPLC-grade methanol (25 mL). Methanol was used for the reconstituted sample dilution to assess Sm content (µg g^−1^ DW) via the HP 1100 Liquid chromatography (Thermo Fisher Scientific, Waltham, MA, USA). The Sm content was expressed as µg g^−1^ DW.

### 2.8. Evaluation of Antioxidant Enzymes

Leafy samples (from all treatments, 0.5 g) were extracted as described by Mukherjee and Choudhuri [42]. After centrifuging the homogenates (15,000× *g*, 10 min), enzyme activities were estimated using the obtained supernatants. SOD activity was measured by monitoring the inhibition of photochemical reduction of nitro-blue tetrazolium (NBT) at 560 nm according to the method described by Giannopolitis and Ries [67]. The activity was determined in a solution containing 50 μM NBT, 1.3 μM riboflavin, 13 mM methionine, 75 nM EDTA, 50 mM phosphate buffer (pH 7.8), and 50 μL enzyme extract. The reaction was started by turning the fluorescent lamps on and stopping 5 min later by turning them off. The blue formazan produced by NBT photoreduction was measured as an increase in absorbance at 560 nm. The reaction mixture lacking leaf extract was taken as control and kept in light. One unit of SOD was defined as the amount of enzyme required to cause 50% inhibition of the rate of NBT reduction at 560 nm compared to the control. The guaiacol oxidation method [68] was used for estimation of POD activity. The reaction mixture (3 mL) contained 0.1 mL enzyme extract, 50 mM phosphate buffer (pH 7.0), 20 mM guaiacol, and 40 mM H_2_O_2_. The change in absorbance of the resulting mixture was read at 470 nm after every 20 s for 180 s. Catalase activity (CAT) was determined using the method of Chandlee and Scandalios [69] with some modifications [64]. Reaction solution (3 mL) of CAT comprised 50 mM phosphate buffer (pH 7.8), 0.1 mL enzyme extract, and 5.9 mM H_2_O_2_ to initiate the reaction. For three min, after every 20 s, CAT activity was noted by measuring the decrease in absorbance at 240 nm using a spectrophotometer. One unit of POD activity was defined as an absorbance change of 0.01 units per min. The APX activity was determined following the method of Asada and Takahashi [70]. The reaction mixture (1600 µL) contained 50 mM potassium phosphate buffer (pH 7.0), 0.5 mM ascorbic acid, 0.1 mM H_2_O_2_, and 400 µL enzyme extract. The absorbance of the mixture was read at 290 nm against the blank, and enzyme activity was expressed in units mg^−1^ protein. The method by Bradford [71] was applied to measure protein content.

### 2.9. Extraction and Evaluation of Phytohormone Levels

Phytohormones levels were assessed in the first completely growing upper leaf (without midribs) on each plant by HPLC apparatus. The chosen leaf from each plant was assigned as a biological replicate, and three independent extractions were performed for each leaf in each treatment. Concisely, 0.2 g of fresh leaf was extracted in 2 mL of distilled water and centrifuged at 12,500 rpm at 4 °C. The pH of the recovered supernatants was adjusted to 2.8–3.2 using acetic acid (30%). The acidified water extract was partitioned twice against 2 mL of diethyl ether. The organic layer was recovered and evaporated under vacuum in a centrifuge concentrator (Speed Vac, Jouan). The dry residue was then suspended in water:methanol (9:1) solution. The resulting solution was filtered and directly injected into an HPLC system (Waters Alliance 2695, Waters Corp.). Utilizing MeOH, the separation for indole-3-acetic acid (IAA) and gibberellins (GAs) was done [72]. After excluding midribs, leaves were frozen in liquid N and then ground. After that, extraction of cytokinins was performed and then analyzed following the procedures of Novák et al. [73]. The SA concentration was determined (ng g^−1^ FW) in the third fully-expanded leaf from the top of each plant in each replicate (pot) following the methods of Siegrist et al. [74] and Metwally et al. [75] using an HPLC system equipped with a fluorescence detector (LC-2010 AHT; Shimadzu, Tokyo, Japan). Abscisic acid (ABA) was measured via HPLC as described by Zeevaart [76] in the same tissue. Frozen samples in triplicates were extracted with 10 mL of extraction buffer (80 mL acetone, 1 mL glacial acetic acid, and 100 mg of 2,6 di-tert-butyl 4-methyl phenol), and collected in a 100 mL volumetric flask. A 2.5 mL plastic syringe with a 0.22 μm filter (Himedia, Delhi, India) was used for samples filtration. An adaptable wavelength 150 UV–vis detector (VWD) and rheodyne injector were used (20 μL loop). The stationary column phase was μBondapak TM/C18 P/N 841/6 S/N with mobile phase 1% (*v*/*v*) acetic acid in 95% methanol solution affixed to the pump. The flow rate was 2.5 mL/min. The wavelength was adjusted to 265 nm before injecting the sample. Determination of ABA in the sample was done by calculating the area under the ABA peak by the specific formula described in the instrument’s principle (A = ½ b × h). Then, compare the area with the standard curve prepared with already known concentrations of ABA.

### 2.10. Molecular Analysis and Gene Expression

#### 2.10.1. Isolation of Total RNA and Synthesis of cDNA

RNA was isolated from control (untreated) and treated chili pepper leaf samples using TRIzol solution and cDNA synthesized in MJ 200CT PCR system using RevertAid™ First Strand cDNA Synthesis Kit (Thermo Scientific), as depicted by AL-Taweel et al. [33].

#### 2.10.2. cDNA-SCoT Amplification and Detection

The PCR reaction was implemented in 25 μL using six SCoT primers (Table 2) for expression profiling study. The concentration of cDNA was adjusted to 40 ng with 30 pmol of primer for PCR amplification. PCR was performed in MJ 200CT Thermal Cycler and gel electrophoresis carried on a BioRad gel system as depicted by AL-Taweel et al. [33]. The banding patterns were photographed using the JSC Gel Documentation.

#### 2.10.3. Patterns of Banding Related to the Expression of cDNA-SCoT

Patterns of banding associated with the expression of cDNA-SCoT were analyzed using CLIQS Gel Image Analysis Software (TotalLab Ltd). Visible transcript-derived amplicons that were identified by analysis of cDNA-SCoT were scored as 0 and 1 matrix. From this matrix, the cDNA-SCoT profile was performed according to Adhikari et al. [77]. With color discrimination for the three types of cDNA-amplicons: cDNA-amplicons that enhanced their genes by one or more treatments (EcDNA-amplicons), cDNA-amplicons that silenced their genes by one or more treatments (ScDNA-amplicons), and monomorphic cDNA-amplicons with control (McDNA-amplicons) to evaluate SCoT-primers efficiency, polymorphic information content (PIC) calculated according to Gorji et al. [78] and resolving power (Rp) were calculated according to Prevost and Wilkinson [79]. The intensities of monomorphic cDNA-amplicons with control were measured using CLIQS Gel Image Analysis software for comparison on its basis among treatments and control.

### 2.11. Data Analysis

After testing for homogeneity of error variances of the three trials data, all experimental data were analyzed applying ANOVA. Fisher’s LSD test was used to identify whether the differences between means (±SE) of all treatments were significant. The analysis software (SAS ver. 9.2; SAS Institute, Inc., Cary, NC, USA) was applied, and differences were identified at *p* ≤ 0.05.

## 3. Results

### 3.1. Growth and Fruit Yield Parameters

Under normal or stress conditions, growth parameters and fruit yield of chili pepper plants were improved by foliar spraying with HB and further improved with HB-Sm, compared to the corresponding control (Table 3).

Shoot fresh weight (ShFW), shoot dry weight (ShDW), root fresh weight (RFW), root dry weight (RDW), number of fruits per plant (NFpP), fresh fruit weight (FFW), and dry fruit weight (DFW) were significantly reduced by salinity by 58.00, 53.78, 51.92, 59.06, 47.56, 51.35, and 61.11%, respectively. Under salt-free conditions, HB or HB-Sm enhanced ShFW, ShDW, RFW, and RDW by 5.54 or 12.56%, 11.63 or 17.13%, 20.85 or 33.62%, and 14.17 or 29.92%, respectively, while under salinity conditions, these growth traits were enhanced by 50.15 or 128.15%, 48.71 or 112.07%, 34.51 or 101.77%, and 55.77 or 140.38%, respectively, compared with the corresponding control. Besides, HB or HB-Sm improved NFpP, FFW, and DFW by 6.10 or 14.63%, 4.05 or 7.43%, and 12.96 or 31.48%, respectively, under salt-free conditions, while under salinity conditions, these yield traits were enhanced by 41.86 or 81.40%, 50.00 or 100.00%, and 95.24 or 147.62%, respectively, compared to the corresponding control. In general, the best growth and fruit yield parameters were gained with foliar nourishment of chili pepper plants with HB-Sm under both salt-free and salinity conditions compared to their controls (Table 3).

### 3.2. Photosynthetic Efficiency

Under normal or stress conditions, the photosynthesis efficiency parameters of chili pepper plants were improved by foliar spraying with HB and further improved with HB-Sm compared to the corresponding control (Table 4).

T. Chls, T. Carot, iEC, Fv/Fm, and photochemical activity (PhA) were significantly reduced by salinity by 60.89, 43.55, 53.85, 46.34, and 45.98%, respectively. However, HB or HB-Sm enhanced T.Chls, T.Carot, iEC, Fv/Fm, and PhA by 8.47 or 17.74%, 11.29 or 25.81%, 15.38 or 26.92%, 6.10 or 8.54%, and 11.38 or 22.10%, respectively, under salt-free conditions, while under salinity conditions, these photosynthetic efficiency attributes were enhanced by 97.74 or 146.39%, 45.71 or 97.14%, 75.00 or 108.33%, 65.91 or 81.82%, and 52.07 or 79.75% with foliar spray by HB or HB-Sm, respectively, compared with the corresponding control. The photosynthetic efficiency was generally maximized with foliar spraying of chili pepper plants with HB-Sm under both salt-free and salinity conditions compared to the corresponding controls (Table 4).

### 3.3. Leaf Relative Water Content (RWC) and Osmoprotectant Contents

Under normal or stress conditions, except for the reduced choline content, RWC and levels of proline, total soluble sugars (TS sugar), and glycine betaine (GB) of the chili pepper plant were significantly increased by foliar spraying with HB and further increased with HB-Sm, compared to the corresponding control (Table 5).

Under salt-free conditions, HB or HB-Sm enhanced the levels of RWC, proline, TS sugar, and GB by 8.90 or 15.04%, 12.31 or 34.39%, 28.46 or 56.10%, and 21.41 or 42.57%, respectively, while under salinity conditions, these parameters were enhanced by 49.15 or 66.10%, 25.12 or 39.21%, 15.09 or 30.66%, and 11.53 or 24.97%, respectively, compared with the corresponding control. Conversely, HB or HB-Sm reduced choline content by 11.0 or 21.64% and 18.60 or 36.57% under salt-free and salinity conditions, respectively, compared with the corresponding control. The best results were generally obtained with foliar nourishment of chili pepper plants with HB-Sm under both salt-free and salinity conditions compared to the corresponding control (Table 5).

### 3.4. K^+^, Na^+^, and Cl^−^ Ions Contents, and the Ratio of K^+^/Na^+^

Under normal or stress conditions, K^+^ content and K^+^/Na^+^ ratio of chili pepper plants were notably enhanced by foliar spraying with HB and further improved with HB-Sm compared with the corresponding control, and vice versa for Na^+^ and Cl^−^ contents (Table 6).

Salinity stress reduced K^+^ content and K^+^/Na^+^ ratio by 57.02 and 98.54%, respectively, while significantly increasing Na^+^ and Cl^−^ contents. Under salt-free conditions, HB or HB-Sm improved K^+^ content and K^+^/Na^+^ ratio by 11.57 or 23.14%, and 23.15 or 57.67%, respectively, while under salinity conditions, these parameters were enhanced by 103.85 or 130.77%, and 436.36 or 2000%, respectively, compared with the corresponding control. Under salt-free conditions, HB or HB-Sm reduced Na^+^ and Cl^−^ contents by 9.38 or 21.88%, and 2.70 or 14.86%, respectively, while these Na^+^ and Cl^−^ contents were decreased under salinity conditions by 63.62 or 89.43%, and 67.03 or 87.97%, respectively, compared with the corresponding control. In general, the highest K^+^ content and K^+^/Na^+^ ratio and minimized Na^+^ and Cl^−^ contents were obtained with foliar nourishment of chili pepper plants with HB-Sm under both salt-free and salinity conditions, compared to the corresponding control (Table 6).

### 3.5. Oxidative Stress Marker Levels and Membrane Damages Related to Oxidative Stress

Under normal or stress conditions, in contrast to the observed elevation of SI, oxidative stress marker levels and membrane damage related to the oxidative stress of chili pepper plants were reduced notably by foliar spraying with HB and further reduced with HB-Sm, compared to the corresponding control (Table 7).

Under salt-free conditions, HB or HB-Sm suppressed the O_2_**^•^^‒^**, H_2_O_2_, MDA, and IL levels by 12.73 or 25.45%, 12.50 or 35.42%, 22.22 or 38.89%, and 12.07 or 22.13%, respectively, while under salinity conditions, these parameters were reduced by 36.44 or 49.15%, 47.39 or 62.02%, 26.19 or 47.62%, and 62.24 or 70.93%, respectively, compared with the corresponding control.

Conversely, under salt-free conditions, HB or HB-Sm enhanced SI by 8.13 or 15.45%, respectively, while under salinity conditions, SI was improved by 56.77 or 88.28%, respectively, compared with the corresponding control. In general, applying HB-Sm maximized the SI level and minimized O_2_^•^**^‒^**, H_2_O_2_, MDA, and IL levels under both salt-free and salinity conditions, compared to the corresponding control (Table 7).

### 3.6. Non-Enzymatic Antioxidant Contents

Under normal or stress conditions, non-enzymatic antioxidant levels of chili pepper plants were enhanced by foliar spraying with HB and further enhanced with HB-Sm, compared to the corresponding control (Table 8).

Under salt-free conditions, HB or HB-Sm enhanced AsA, GSH, ToC, phenolics, and Sm levels by 11.88 or 23.75%, 18.32 or 34.35%, 2.60 or 21.86%, 0.24 or 3.59%, and 12.99 or 26.77%, respectively, while under salinity conditions, these AsA, GSH, ToC, phenolics, capsaicin, and Sm levels were enhanced by 12.25 or 21.32%, 17.17 or 32.32%, 10.58 or 22.18%, 9.96 or 20.31%, 12.77 or 29.79%, and 14.89 or 36.70%, respectively, compared with the corresponding control. In general, the highest AsA, GSH, ToC, phenolics, capsaicin, and Sm contents were obtained with foliar nourishment of the chili pepper plants with HB-Sm under both salt-free and salinity conditions compared to the corresponding control (Table 8).

### 3.7. Enzymatic Antioxidant Activities

Under normal or stress conditions, enzymatic antioxidant activities of chili pepper plants were enhanced by foliar spraying with HB and further enhanced with HB-Sm compared to the corresponding control (Table 9).

Under salt-free conditions, HB or HB-Sm enhanced SOD, CAT, POD, and APX activities by 10.56 or 21.74%, 23.89 or 55.47%, 15.75 or 28.77%, and 13.93 or 29.51%, respectively, while under salinity conditions, the activities were enhanced by 8.64 or 18.86%, 18.92 or 41.16%, 14.41 or 34.23%, and 15.91 or 40.91%, respectively, compared to the corresponding control. In general, the highest activities of all enzymes were collected with foliar nourishment of the chili pepper plants with HB-Sm under both salt-free and salinity conditions, compared to the corresponding control (Table 9).

### 3.8. Hormonal Content

Under normal or stress conditions, in contrast to the observed reduction of ABA content; IAA, GAs, cytokinin, and salicylic acid (SA) levels were enhanced by foliar spraying with HB and further enhanced with HB-Sm, compared to the corresponding control (Table 10).

The contents of IAA, GAs, and cytokinin were significantly reduced by salinity by 41.77, 51.09, and 50.0%, respectively, and vice versa for SA and ABA contents, which were increased by 33.78 and 150.39%, respectively. Under salt-free conditions, HB or HB-Sm enhanced the levels of IAA, GAs, cytokinin, and SA by 8.86 or 10.76%, 10.90 or 15.26%, 15.12 or 32.56%, and 14.19 or 29.73%, respectively, while under salinity conditions, these hormones were enhanced by 57.61 or 75.00%, 79.62 or 102.55%, 60.47 or 96.51%, and 14.14 or 28.79%, respectively compared with the corresponding control. In contrast, HB or HB-Sm reduced ABA content by 36.91 or 58.20%, respectively, under salt-free conditions, while under salinity conditions, ABA content was decreased by 51.33 or 59.59%respectively, compared with the corresponding control. In general, the highest hormonal (IAA, GAs, and cytokinin) levels and minimal ABA level were obtained with foliar nourishing of chili pepper plants with HB-Sm under both salt-free and saline conditions, compared to the corresponding control (Table 10).

### 3.9. Differential Expression Analysis Using the cDNA-SCoT Technique

**Figure 1:**
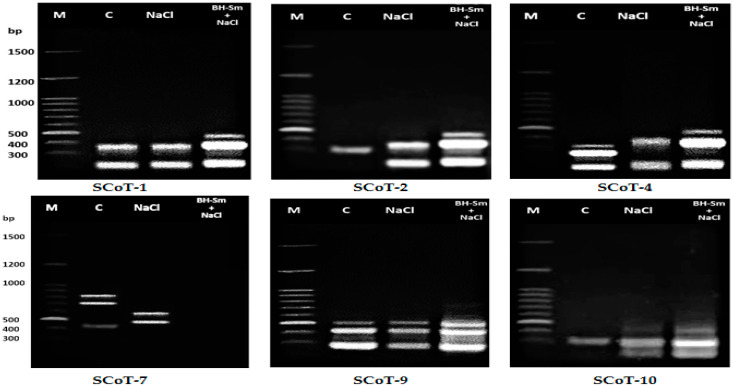
cDNA-SCoT amplification of SCoT-1, SCoT-2, SCoT-4, SCoT-7, SCoT-9, and SCoT-10 of chili pepper after treatments with NaCl and HB-Sm compared with the control.

**Figure 2:**
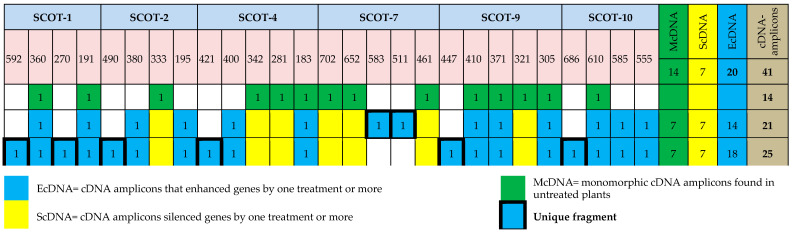
The DNA profile of cDNA amplicons resulting from the cDNA-SCoT technique. cDNA amplicons: transcript-derived amplicons ScDNA: transcript-derived amplicons whose silenced their genes by one or more treatments; EcDNA: transcript-derived amplicons whose enhanced their genes by one or more treatments; McDNA: monomorphic cDNA-amplicons with control.

Molecular banding patterns and DNA profiles based on cDNA-SCoT are presented in Figure 1 and Figure 2, which proved that gene pattern expression differed between the treated chili pepper with HB-Sm and the untreated ones by targeting 41 recordable cDNA amplicons (average of 6.83 cDNA amplicons/primer). The product size was 191–702 bp. Of these cDNA amplicons, 14 appeared in the untreated chili pepper plant, while the treated ones had 7 of 14 cDNA-amplicons. Of the 41 cDNA amplicons centered using SCoT primers, 20 were EcDNA amplicons (unique transcripts) with 49%, while the ScDNA amplicons, which silenced genes in one or more treatments, were 7 out of 14 McDNA amplicons with 17%. Table 11 revealed an equal number of amplicons exhibited by SCoT-4, SCoT-7, SCoT-9 (5 amplicons), Scot-1, SCoT-2, and SCoT-10 (4 amplicons). On the other hand, SCoT-4 showed the lowest number of EcDNA amplicons (3 amplicons). SCoT-9 recorded a high Rp (4.44) value in line with 82.4% polymorphism and 0.311 of PIC.

## 4. Discussion

In arid and semi-arid areas, the agricultural sector suffers from numerous stressors. Researchers are trying to find solutions to obtain yields commensurate with the considerable efforts to maintain sustainable agricultural development [7,18,19,80,81,82,83,84]. Amongst stressors, salinity often compromises plant performance and its returns (yields) through salt influences on different processes related to plant metabolism by inducing the immoderate generation of reactive oxygen species (ROS), seriously influencing plant growth and development [85,86]. To avert salt damage, stressed plants stimulate several specific strategic mechanisms, including osmotic adjustment, ion hemostasis, and stimulation of antioxidant defense components [7,87]. However, plants’ self-defenses cannot be sufficient under prolonged stress. Therefore, plants must be supported by exogenous applications [7,18,19,23,57,85,88,89]. Recently, diluted honey bee (HB) was used alone as a highly efficient multi-biostimulant [22].

HB displays an efficient role in mitigating the stress impacts in plants due to its richness in various nutrients (i.e., K, Mg, Ca, P, Fe, Mn, Zn, Cu, and I), osmotic compounds (i.e., soluble sugars and free proline), and antioxidant compounds (i.e., ascorbic acid; vitamin C) (Table 1). In agreement with Semida et al. [22], our results confirmed that HB is a potent biostimulant of growth and a rich nutritional substance that protects chili pepper plants from salt stress influences due to the high antioxidant activity (88.2%) and B-group vitamins, which are crucial focal mechanisms to prohibit oxidation of membrane lipids and increase tissue cell water content and minimize the suppression of cell oxidative stress damage under stress [22].

On the other hand, Alharby et al. [18] recently explored the role of silymarin (Sm) as an auxiliary metabolite to increase plant performance under abiotic stresses due to the contribution of Sm in improving the defense system of stressed plants. Therefore, our research is the first in which HB enriched with silymarin (HB-Sm; a multi-biostimulator) was applied as foliar nourishment to encourage the growth of chili pepper plants under NaCl-salinity stress. HB-Sm positively affected growth parameters, metabolism, components of the antioxidant defense system, and fruit yield under normal and salinity conditions due to the rise in plant resistance to stress (Table 12). This finding is in agreement with those obtained by Rosa [90], Alharby et al. [18], and Semida et al. [22], who confirmed that biostimulants, including HB and Sm, stimulate plant tolerance against stress damage. Our results demonstrated that the bioactive components of HB-Sm, including Sm, osmoprotectants (proline and soluble sugars), organic nutrients, and antioxidants [ascorbate (AsA) and B-group vitamins] promoted the plant’s ability to adapt to stress adverse conditions by improving plant metabolism, cell elongation, and division, and dry matter accumulation, thus allowing plants to resist the harmful impacts of stress, greatly enhancing plant performance and yield [18,22].

Our study clarified that plant growth (shoot and root fresh and dry weights) and yield (number of fruits per plant, fresh and dry fruit weights) were significantly enhanced by foliar spray with HB and further enhanced by using HB-Sm under salt-free and salinity conditions. It indicates that the enhanced root growth helped the plants to withstand stress conditions. These positive effects are generated through different signaling pathways that influence adaptive plant responses to environmental stresses and/or the regulation and expression of genes that contribute to tolerance and are stimulated by stress [90]. Biostimulants increase plant roots and shoots’ growth, raise photosynthetic efficiency and water uptake, helping to increase resistance to stress [18,19,22,24,38,91,92,93]. In biostimulator-treated plants, stimulation of metabolic pathways combined with phenylpropanoid synthesis may explain the attenuation of stress effects in plants [24].

The enhanced growth traits in our results by HB-Sm may be due to its richness in essential organic nutrients, proline, and soluble sugars, which are vital for cellular protoplasm formation [94]. HB-Sm also contains various phytohormones, including cytokinin and auxins (Table 1), responsible for rapid cell enlargement, division, and multiplication [22]. Besides, HB increases the antioxidant capacity, and Sm gives plants additional antioxidant ability to attenuate salt stress influences [18,22,85,95]. The antioxidants available in HB-Sm (e.g., Sm, AsA, proline, and group B vitamins) penetrated plant leaves through foliar spraying and regulated the plant’s adaptive response to stresses, reflecting plant growth and development [18,22].

Optimization of photosynthesis by application of HB or HB-Sm resulted in additional photosynthetic substances, including soluble sugars, free proline, etc., besides those infiltrated in plants by spraying of HB or HB-Sm (Table 5 and Table 12). This result could contribute to osmotic adjustment, cell integration, elevated RWC, and SI (Table 5, Table 7 and Table 12). Besides, chlorophyll content and performance were significantly promoted by HB or HB-Sm and delayed leaf senescence (data not shown). Under salt stress, plant metabolism was enhanced due to the protective impacts of HB or HB-Sm on different photosynthetic systems. The results indicate a robust correlation between the increased antioxidant and osmoprotective compounds (Table 5, Table 8 and Table 12) and the capacity of salt-stressed plants to survive [86,96,97]. This report confirmed that proline acts as an effective free radical scavenger, protecting enzymes and/or like-minded solute causation in osmotic modifications. Also, Rosa et al. [90] mentioned that the main group of compatible organic solutes, soluble sugars, serve as a master role in alleviating abiotic stress significantly by osmotic alteration, which is a vital mechanism involved in plant adaptations for many stressors [96,97,98]. Our results signalize that the water status of chili pepper plants depends mainly on root and shoot biomass. A plant with higher biomass can retain higher water and become more tolerant to salinity stress. RWC vs. plant biomass can be used as a part of an appropriate signal to discriminate the specific and non-specific characters for salt stress tolerance in chili pepper plants [93]. This report signalizes a strong relationship between plant biomass accumulation and tissue RWC under stress due to antioxidant applications.

HB-Sm significantly reduced oxidative stress marker levels and membrane damage related to oxidative stress in chili pepper plant compared to the control (Table 7 and Table 12). The reports [18,22] confirm our results. Non-enzymatic antioxidant contents of chili pepper plants were enhanced under saline conditions and further enhanced by foliar spraying with HB and HB-Sm (Table 8 and Table 12). HB-Sm improved capsaicin content under saline conditions. However, the highest contents of AsA, GSH, ToC, phenolic compounds, and Sm were obtained by HB-Sm under both salt-free and salinity conditions compared to the controls. These increased antioxidants help chili pepper plant resist salinity stress by accounting for several mechanisms with other bioactive plant components, along with morphological and structural changes and increased secondary products [4,18,22,99]. Besides, higher contents of the osmoprotective compounds in chili pepper plants by HB or HB-Sm application promoted green photosynthetic pigments to induce carbohydrate metabolism, creating new potent relationships between sources and increasing plant dry matter accumulation to counteract stressful conditions [97]. The increase in plant antioxidants (e.g., AsA, GSH, proline, ToC, capsaicin, phenols, and Sm) occurred in parallel with the enhancement in antioxidant enzyme activities (e.g., SOD, CAT, POD, and APX) in chili pepper plants sprayed with HB and these improvements were even more with HB-Sm under both salt-free and salinity conditions (Table 8, Table 9 and Table 12). These enhanced enzymatic and non-enzymatic antioxidants supported the plant’s antioxidant capacity to withstand stress in plants [4,18,19,22].

In this study, except for the decrease in ABA, phytohormone (IAA, GAs, and cytokinin) contents were significantly enhanced in chili pepper plants by foliar spraying with HB and further enhanced with HB-Sm under salinity stress (Table 10 and Table 12) to enable plants to withstand stress [22,100]. These phytohormones participate with antioxidants in attenuating and repairing ROS damage under stress by supporting plants with a complex antioxidative defense system [22,101,102]. Diluted HB catalyzes glucose oxidation by glucose oxidase to form H_2_O_2_ at low concentrations [22], which is protective in relieving abiotic stresses, including salt stress [87,103]. Besides, Sm accumulates in stressed plants to encourage their defense systems to enhance performance and productivity [18,26]. In this study, the bioactive ingredients of HB-Sm, including Sm and low H_2_O_2_ level, greatly enhanced its efficiency in increasing the chili pepper plant’s tolerance to NaCl-salinity stress to perform well under salt stress.

Our investigation applied cDNA-SCoT based techniques to analyze gene expression variations induced by HB-Sm applications. cDNA-SCoT could be readily used to investigate the total level of plant transcripts simultaneously, which is particularly important for analyzing differential expression [104]. Six SCoT primers, which target genes expressing high profile, were used to investigate the effect of HB-Sm treatments on pattern expression of the gene [105] for the targeted amplification of cDNA derived from RNA extracted from treated and untreated chili pepper plants. From the cDNA amplicons, 14 appeared in the untreated plants and seven cDNA amplicons in treated plants. Our results are consistent with those obtained by Al-Taweel et al. [33,106] on the response of stevia plants to different salt concentrations.

In contrast, cDNA-SCoT markers produced different amplicons sizes in treated plants and were absent in untreated plants (control). Of 41 cDNA amplicons revealed by SCoT primers, 20 were EcDNA amplicons (cDNA-amplicons that enhanced their genes by one or more treatments) with 49% of total amplicons. At the same time, the ScDNA amplicons that silenced genes in one or more treatments were 7 amplicons with 17% out of total amplicons, while the McDNA (monomorphic cDNA-amplicons with control) was presented by 14 amplicons with 34% of total amplicons. The HB-Sm treatment has been highly influential in augmenting the expression of some genes in untreated plants, also silencing the expression of active genes aimed by SCoT primers in untreated plants. Wu et al. [104] applied the cDNA-SCoT-based approach to detect gibberellin-induced differential express genes during sugarcane elongation and gene downregulation and upregulation.

He et al. [107] reported that transcript silencing, which could be reversible, resulted in a loss of gene or expression repressing, which was reported irreversible. As we found, one or more treatments resulted in losing transcripts (7 ScDNA out of 41 by 17%) (Figure 1 and Figure 2). Our findings demonstrate the highly discriminatory ability and information of SCoT-9 in detecting differentially expressed genes between control and treated plants, compared to others. The results also confirmed the success of cDNA-SCoT in detecting patterns of variability expression of the gene among treated and untreated plants by HB-Sm [105,107]. Also, Abd El-Maksoud et al. [108] illustrated the same results among wheat seedlings exposed to drought stress.

The major benefits of diluted HB, as reviewed in [109], can also be attributed to the chemical composition of honey colloids comprising markedly higher levels of minerals such as Fe, Si, Ca, and Mg, as well as relatively high molecular weight proteins, pentosans (pentose polymers), nitrogenous compounds, and inorganic substances. It also contains phenolic acids, flavonoids, high antioxidants, and antibacterial activities. Besides, the enzyme activity in honey colloids may be affected by concentration. It may be altered concerning (a) the antioxidant activity of honey polyphenols during the formation of melanoidins, (b) the enzymatic production of H_2_O_2_, and (c) the antibacterial activity upon honey dilution. An interesting finding, in this study, is that the reduced H_2_O_2_ concentration in diluted HB contributed to the reduced level of H_2_O_2_ in stressed chili pepper plants (Table 7 and Table 12). Brudzynski [110] added that H_2_O_2_ production by glucose oxidase is active in diluted honey. Several studies have shown that honey’s growth-inhibitory and bactericidal activities are strongly influenced by H_2_O_2_ concentration [109,110,111]. Glucose oxidation is the main pathway of H_2_O_2_ production in honey, although other mechanisms are now known [110]. There is a strong relationship between H_2_O_2_ levels and the antibacterial activity of honey [22,112]. Further studies are needed to demonstrate the role of H_2_O_2_ levels of diluted HB and antioxidant activity of Sm in enhancing plant tolerance to stress.

## 5. Conclusions

The present study’s findings demonstrate that exogenously-applied honey bee enriched with silymarin (HB-Sm) was the best treatment, which effectively attenuated salinity (10 dS m^‒1^ using NaCl salt) stress damage to the chili pepper plant growth, physiological, and yield attributes through the increase in K^+^/Na^+^ ratio, non-enzymatic antioxidant, and osmoprotectant levels, enzymatic antioxidant activities, hormonal contents, and gene expressions along with decreased Na^+^ and Cl^‒^ contents, oxidative stress markers, and ABA levels, and oxidative stress-related membrane damage. These positive findings are ascribed to the bioactive components (e.g., sugars, proline, organic nutrients, and vitamin C) and high antioxidant activity (88.2%) of HB-Sm, playing a crucial role in plant development and metabolism, and plant response to salt stress. Therefore, HB-Sm can be used as an inexpensive biostimulant and/or micro-elements nutrient source for plants under normal or abnormal conditions as an alternative to expensive synthetic substances. More research is imperative and indispensable to explore the precise mechanisms of this promising HB-Sm as a multi-stimulator in signaling pathways and physiological responses to abiotic stresses.

## Figures and Tables

**Table 1:** Major ingredients of raw clover honey (based on fresh weight).

Component	Unit	Value	Reference
Moisture	%	17.8	[39]
pH	-	3.8	
**Osmoprotectants：**			
Proline	μmol kg^−1^ FW	359.8	[39]
Total soluble sugars	%	82.0	
**Sugar fractions：**			
Fructose	μmol kg^−1^ FW	212.0	[40]
Glucose		168.7	
Maltose		12.0	
Sucrose		19.0	
**Mineral nutrients:**			
Potassium (K)	mmol kg^−1^ FW	7.98	[41]
Magnesium (Mg)		3.14	
Calcium (Ca)		1.68	
Phosphorus (P)		1.04	
Iron (Fe)		0.99	
Manganese (Mn)		0.84	
Zinc (Zn)		0.08	
Copper (Cu)		0.06	
Iodine (I)		0.62	
**Antioxidants:**			
Ascorbic acid (AsA; Vitamin C)	μmol kg^−1^ FW	72.2	[42]
DPPH radical-scavenging activity	%	88.2	[43]

**Table 2:** SCoT primer sequences.

SCoT Primers	Sequence (5′-3′)	%GC	Molecular Size Range
1	CAACAATGGCTACCACCA	50	191:592
2	CAACAATGGCTACCACCC	56	395:680
4	CAACAATGGCTACCACCT	50	183:421
7	CAACAATGGCTACCACGG	56	461:702
9	CAACAATGGCTACCAGCA	50	281:640
10	CAACAATGGCTACCAGCC	56	555:762

**Table 3:** Response of growth and yield traits of salt-stressed chili pepper to foliar spray with bee-honey (HB) supplemented with silymarin (HB-Sm).

**Treatment**	**Growth Characteristics**					
	**Shoot FW** **(g plant^−1^)**	**Shoot DW** **(g plant^−1^)**		**Root FW** **(g plant^−1^)**		**Root DW** **(g plant^−1^)**
Control	81.2 ± 5.1 ^b^	50.2 ± 3.2 ^b^		23.5 ± 2.1 ^c^		12.7 ± 1.1 ^c^
HB	85.7 ± 5.9 ^ab^	56.04 ± 0.51 ^ab^		28.4 ± 3.5 ^b^		14.5 ± 0.33 ^b^
HB-Sm	91.4 ± 6.2 ^a^	58.8 ± 0.55 ^a^		31.4 ± 3.8 ^a^		16.5 ± 0.32 ^a^
Salinity (S)	34.1 ± 2.4 ^d^	23.2 ± 0.22 ^d^		11.3 ± 1.9 ^e^		5.2 ± 0.18 ^e^
S + HB	51.2 ± 3.7 ^c^	34.5 ± 0.38 ^c^		15.2 ± 2.7 ^d^		8.1 ± 0.22 ^d^
S + HB-Sm	77.8 ± 4.9 ^b^	49.2 ± 0.44 ^b^		22.8 ± 3.2 ^c^		12.5 ± 0.22 ^c^
	**Yield Parameters**					
	**No. of fruits plant^−1^**		**Fresh fruit weights (g plant^−1^)**		**Dry fruit weights (g plant^−1^)**	
Control	8.2 ± 0.7 ^b^		14.8 ± 1.5 ^b^		5.4 ± 0.3 ^c^	
HB	8.7 ± 0.9 ^ab^		15.4 ± 1.5 ^ab^		6.1 ± 0.4 ^b^	
HB-Sm	9.4 ± 1.0 ^a^		15.9 ± 1.6 ^a^		7.1 ± 0.6 ^a^	
Salinity (S)	4.3 ± 0.5 ^d^		7.2 ± 0.8 ^d^		2.1 ± 0.2 ^e^	
S + HB	6.1 ± 0.6 ^c^		10.8 ± 0.9 ^c^		4.1 ± 0.3 ^d^	
S + HB-Sm	7.8 ± 0.8 ^b^		14.4 ± 1.2 ^b^		5.2 ± 0.4 ^c^	

Means (±SE) in each column followed by different letters are significant at the *p* ≤ 0.05 level based on Fisher’s least significant difference (LSD) tests. Growth characteristics were measured in 50 days after transplanting (DAT) samples, while yield parameters were assessed at harvest (95 DAT). FW = fresh weight and DW = dry weight.

**Table 4:** Response of photosynthesis efficiency of salt-stressed chili pepper to foliar spray with bee-honey (HB) supplemented with silymarin (HB-Sm).

Treatment	T. Chls (mg g^−^^1^ FW)	T. Carot (mg g^−^^1^ FW)	iCE (µmol m^−^^2^ s^−^^1^)	Fv/Fm	PhA
Control	2.48 ± 0.06 ^c^	0.62 ± 0.02 ^c^	0.26 ± 0.01 ^c^	0.82 ± 0.02 ^b^	44.8 ± 0.8 ^c^
HB	2.69 ± 0.08 ^b^	0.69 ± 0.02 ^b^	0.30 ± 0.01 ^b^	0.87 ± 0.03 ^a^	49.9 ± 1.1 ^b^
HB-Sm	2.92 ± 0.09 ^a^	0.78 ± 0.03 ^a^	0.33 ± 0.01 ^a^	0.89 ± 0.03 ^a^	54.7 ± 1.4 ^a^
Salinity (S)	0.97 ± 0.03 ^e^	0.35 ± 0.01 ^e^	0.12 ± 0.00 ^e^	0.44 ± 0.01 ^d^	24.2 ± 0.3 ^e^
S + HB	1.92 ± 0.04 ^d^	0.51 ± 0.01 ^d^	0.21 ± 0.01 ^d^	0.73 ± 0.02 ^c^	36.8 ± 0.6 ^d^
S + HB-Sm	2.39 ± 0.05 ^c^	0.69 ± 0.02 ^b^	0.25 ± 0.01 ^c^	0.80 ± 0.02 ^b^	43.5 ± 0.7 ^c^

Means (±SE) in each column followed by different letters are significant at the *p* ≤ 0.05 level based on Fisher’s least significant difference (LSD) tests. PhA means photochemical activity. T. Chls = total chlorophyll, T. Carot = total carotenoids, iCE = instantaneous efficiency of carboxylation, Fv/Fm = Photosystem II quantum efficiency, PhA = photochemical activity.

**Table 5:** Response of leaf relative water content (RWC, %) and osmoprotectant contents of salt-stressed chili pepper to foliar spray with bee-honey (HB) supplemented with silymarin (HB-Sm).

Treatment	RWC (%)	Proline Content (μmol g^−1^ DW)	T.S. Sugars Content (mg g^−1^ DW)	GB Content (μmol g^−1^ DW)	Choline Content (μmol g^−1^ DW)
Control	79.8 ± 5.5 ^b^	94.2 ± 2.5 ^e^	12.3 ± 0.2 ^e^	122.4 ± 3.2 ^f^	198.2 ± 5.2 ^a^
HB	86.9 ± 5.8 ^a^	105.8 ± 2.8 ^d^	15.8 ± 0.3 ^d^	148.6 ± 4.4 ^e^	176.4 ± 4.0 ^b^
HB-Sm	91.8 ± 7.2 ^a^	126.6 ± 2.9 ^c^	19.2 ± 0.4 ^c^	174.5 ± 4.6 ^d^	155.3 ± 4.0 ^b^
Salinity (S)	47.2 ± 3.7 ^d^	129.8 ± 3.2 ^c^	21.2 ± 0.5 ^c^	192.6 ± 5.4 ^c^	129.6 ± 2.6 ^c^
S + HB	70.4 ± 5.2 ^c^	162.4 ± 3.5 ^b^	24.4 ± 0.6 ^b^	214.8 ± 6.7 ^b^	105.5 ± 2.2 ^d^
S + HB-Sm	78.4 ± 6.0 ^b^	180.7 ± 3.8 ^a^	27.7 ± 0.6 ^a^	240.7 ± 7.4 ^a^	82.2 ± 1.4 ^e^

Means (±SE) in each column followed by different letters are significant at the *p* ≤ 0.05 level based on Fisher’s least significant difference (LSD) tests. TS sugars = Total soluble sugars, and GB = Glycine betaine.

**Table 6:** Response of osmoprotectant contents of salt-stressed chili pepper to foliar spray with bee-honey (HB) supplemented with silymarin (HB-Sm).

Treatment	K^+^ Ion Content (mg g^−1^ DW)	Na^+^ Ion Content (mg g^−1^ DW)	K^+^/Na^+^ Ratio	Cl^−^ Ion Content (mg g^−1^ DW)
Control	2.42 ± 0.05 ^c^	0.32 ± 0.01 ^d^	7.56 ± 0.16 ^c^	0.74 ± 0.02 ^d^
HB	2.70 ± 0.06 ^b^	0.29 ± 0.01 ^de^	9.31 ± 0.20 ^b^	0.72 ± 0.02 ^d^
HB-Sm	2.98 ± 0.06 ^a^	0.25 ± 0.01 ^e^	11.92 ± 0.23 ^a^	0.63 ± 0.01 ^e^
Salinity (S)	1.04 ± 0.02 ^e^	9.84 ± 0.18 ^a^	0.11 ± 0.01 ^f^	12.80 ± 0.10 ^a^
S + HB	2.12 ± 0.04 ^d^	3.58 ± 0.05 ^b^	0.59 ± 0.02 ^e^	4.22 ± 0.08 ^b^
S + HB-Sm	2.40 ± 0.05 ^bc^	1.04 ± 0.03 ^c^	2.31 ± 0.05 ^d^	1.54 ± 0.04 ^c^

Means (±SE) in each column followed by different letters are significant at the *p* ≤ 0.05 level based on Fisher’s least significant difference (LSD) tests.

**Table 7:** Response of oxidative stress marker levels and membrane damages related to oxidative stress of salt-stressed chili pepper to foliar spray with bee-honey (HB) supplemented with silymarin (HB-Sm).

Treatment	O_2_^•^^−^ Level (A_580_ g^−1^ FW)	H_2_O_2_ Level (μmol g^‒1^ FW)	MDA Level (A_532–600_ g^−1^ FW)	IL (%)	SI (%)
Control	0.55 ± 0.01 ^d^	1.92 ± 0.03 ^d^	0.18 ± 0.002 ^d^	6.96 ± 0.30 ^c^	73.8 ± 4.4 ^c^
HB	0.48 ± 0.01 ^e^	1.68 ± 0.03 ^e^	0.14 ± 0.002 ^e^	6.12 ± 0.24 ^d^	79.8 ± 4.8 ^b^
HB-Sm	0.41 ± 0.01 ^f^	1.24 ± 0.03 ^f^	0.11 ± 0.001 ^f^	5.42 ± 0.20 ^e^	85.2 ± 5.5 ^a^
Salinity (S)	1.18 ± 0.02 ^a^	5.74 ± 0.09 ^a^	0.42 ± 0.009 ^a^	24.42 ± 1.31 ^a^	38.4 ± 2.2 ^e^
S + HB	0.75 ± 0.02 ^b^	3.02 ± 0.05 ^b^	0.31 ± 0.006 ^b^	9.22 ± 0.44 ^b^	60.2 ± 3.3 ^d^
S + HB-Sm	0.60 ± 0.01 ^c^	2.18 ± 0.04 ^c^	0.22 ± 0.002 ^c^	7.10 ± 0.32 ^c^	72.3 ± 3.8 ^c^

Means (±SE) in each column followed by different letters are significant at the *p* ≤ 0.05 level based on Fisher’s least significant difference (LSD) tests. O_2_^•^^‒^ = superoxide ions, H_2_O_2_ = hydrogen peroxide, MDA = malondialdehyde, IL% = ionic leakage, SI (%) = stability index.

**Table 8:** Response of non-enzymatic antioxidant levels of salt-stressed chili pepper to foliar spray with bee-honey (HB) supplemented with silymarin (HB-Sm).

Treatment	AsA Content (μmol g^−1^ FW)	GSH Content (μmol g^−1^ FW)	ToC (μmol g^−1^ DW)	Phenolic Content (mg kg^−1^ FW)	Capsaicin (mg kg^−1^ DW)	Sm Content (mg kg^−1^ DW)
Control	3.20 ± 0.04 ^e^	1.31 ± 0.02 ^f^	46.2 ± 0.4 ^e^	41.8 ± 0.3 ^e^	0.84 ± 0.02 ^d^	25.4 ± 0.4 ^f^
HB	3.58 ± 0.04 ^d^	1.55 ± 0.02 ^e^	47.4 ± 0.4 ^d^	41.9 ± 0.3 ^e^	0.82 ± 0.02 ^d^	28.7 ± 0.4 ^e^
HB-Sm	3.96 ± 0.05 ^c^	1.76 ± 0.03 ^d^	56.3 ± 0.5 ^c^	43.3 ± 0.3 ^d^	0.85 ± 0.02 ^d^	32.2 ± 0.5 ^d^
Salinity (S)	4.08 ± 0.05 ^c^	1.98 ± 0.03 ^c^	58.6 ± 0.6 ^c^	52.2 ± 0.4 ^c^	0.94 ± 0.03 ^c^	37.6 ± 0.6 ^c^
S + HB	4.58 ± 0.06 ^b^	2.32 ± 0.04 ^b^	64.8 ± 0.6 ^b^	57.4 ± 0.5 ^b^	1.06 ± 0.04 ^b^	43.2 ± 0.6 ^b^
S + HB-Sm	4.95 ± 0.06 ^a^	2.62 ± 0.04 ^a^	71.6 ± 0.6 ^a^	62.8 ± 0.6 ^a^	1.22 ± 0.05 ^a^	51.4 ± 0.8 ^a^

Means (±SE) in each column followed by different letters are significant at the *p* ≤ 0.05 level based on Fisher’s least significant difference (LSD) tests. Capsaicin was determined in fruits, AsA = ascorbate, GSH = glutathione, ToC = α-tocopherol, Sm = silymarin.

**Table 9:** Response of enzymatic antioxidant activities of salt-stressed chili pepper to foliar spray with bee-honey (HB) supplemented with silymarin (HB-Sm).

Treatment	Activity of SOD (Units mg^−1^ Protein)	Activity of CAT (Units mg^−1^ Protein)	Activity of POD (Units mg^−1^ Protein)	Activity of APX (Units mg^−1^ Protein)
Control	32.2 ± 0.48 ^f^	4.94 ± 0.06 ^f^	14.6 ± 0.19 ^f^	12.2 ± 0.15 ^f^
HB	35.6 ± 0.55 ^e^	6.12 ± 0.09 ^e^	16.9 ± 0.28 ^e^	13.9 ± 0.18 ^e^
HB-Sm	39.2 ± 0.58 ^d^	7.68 ± 0.11 ^d^	18.8 ± 0.32 ^d^	15.8 ± 0.20 ^d^
Salinity (S)	44.0 ± 0.64 ^c^	9.62 ± 0.14 ^c^	22.2 ± 0.38 ^c^	17.6 ± 0.25 ^c^
S + HB	47.8 ± 0.74 ^b^	11.44 ± 0.18 ^b^	25.4 ± 0.42 ^b^	20.4 ± 0.30 ^b^
S + HB-Sm	52.3 ± 0.84 ^a^	13.58 ± 0.22 ^a^	29.8 ± 0.48 ^a^	24.8 ± 0.36 ^a^

Means (±SE) in each column followed by different letters are significant at the *p* ≤ 0.05 level based on Fisher’s least significant difference (LSD) tests. SOD = superoxide dismutase, CAT = catalase, POD = peroxidase, APX = ascorbate peroxidases activities.

**Table 10:** Response of hormonal contents of salt-stressed chili pepper to foliar spray with bee-honey (HB) supplemented with silymarin (HB-Sm).

Treatment	IAA Content	GAs Content	Cytokinin Content	Salicylic Acid	ABA Content
	(ng g^−^^1^ FW)				
Control	15.8 ± 0.02 ^b^	32.1 ± 0.05 ^b^	17.2 ± 0.03 ^c^	148 ± 1.8 ^e^	5.12 ± 0.01 ^c^
HB	17.2 ± 0.03 ^a^	35.6 ± 0.06 ^a^	19.8 ± 0.03 ^b^	169 ± 1.9 ^d^	3.23 ± 0.01 ^d^
HB-Sm	17.5 ± 0.03 ^a^	37.0 ± 0.07 ^a^	22.8 ± 0.04 ^a^	192 ± 2.4 ^c^	2.14 ± 0.00 ^e^
Salinity (S)	9.2 ± 0.01 ^d^	15.7 ± 0.02 ^d^	8.6 ± 0.02 ^e^	198 ± 2.5 ^c^	12.82 ± 0.02 ^a^
S + HB	14.5 ± 0.02 ^c^	28.2 ± 0.04 ^c^	13.8 ± 0.03 ^d^	226 ± 2.9 ^b^	6.24 ± 0.01 ^b^
S + HB-Sm	16.1 ± 0.03 ^b^	31.8 ± 0.05 ^b^	16.9 ± 0.03 ^c^	255 ± 3.2 ^a^	5.18 ± 0.01 ^c^

Means (±SE) in each column followed by different letters are significant at the *p* ≤ 0.05 level based on Fisher’s least significant difference (LSD) tests. IAA = indole-3-acetic acid, GAs = gibberellins, ABA = Abscisic acid.

**Table 11:** The six primers revealed patterns of cDNA-SCoT banding.

Primer Name	cDNA Amplicons					P%	PIC	Rp
	bp Range	McDNA	ScDNA	EcDNA	Total			
SCoT-1	191:592	0	2	4	6	100.0	0.387	3.774
SCoT-2	195:490	0	2	3	5	100.0	0.387	3.774
SCoT-4	183:421	3	4	5	12	75.0	0.333	2.220
SCoT-7	461:702	3	6	2	11	72.7	0.375	2.664
SCoT-9	305:447	3	0	14	17	82.4	0.311	4.440
SCoT-10	555:686	1	0	10	11	91.0	0.296	2.220

McDNA = monomorphic cDNA amplicons found in untreated plants, ScDNA = cDNA amplicons that silenced genes by one treatment or more EcDNA = cDNA amplicons that enhanced genes by one treatment or more, P% = Polymorphism%, PIC = Polymorphic index content, Rp = Resolving power.

**Table 12:** Changes (%) in various attributes related to plant morpho-physio-biochemistry corresponding to the control in salt-treated chili pepper plants foliar sprayed with bee-honey (HB) and silymarin (Sm). Three color scale heatmap, red for changes over control values, green for changes below control values, and yellow as the midpoint of control and parameters with insignificant values compared to the control.

Parameters	Treatments					
	Control	HB	HB-Sm	Salinity (S)	S + HB	S + HB-Sm
Shoot FW	81.2 ^b^	+5.54 ^ab^	+12.56 ^a^	−58.00 ^d^	−36.95 ^c^	−4.18 ^b^
Shoot DW	50.2 ^b^	+11.63 ^ab^	+17.13 ^a^	−53.78 ^d^	−31.27 ^c^	−1.99 ^b^
Root FW	23.5 ^c^	+20.85 ^b^	+33.62 ^a^	− 51.91 ^e^	−35.32 ^d^	−2.98 ^c^
Root DW	12.7 ^c^	+14.17 ^b^	+29.92 ^a^	−59.06 ^e^	−36.22 ^d^	−1.57 ^c^
No. of fruits plant^−1^	8.2 ^b^	+6.10 ^ab^	+14.63 ^a^	−47.56 ^d^	− 25.61 ^c^	−4.88 ^b^
Fresh fruit weights	14.8 ^b^	+4.05 ^ab^	+7.43 ^a^	−51.35 ^d^	−27.03 ^c^	−2.70 ^b^
Dry fruit weights	5.4 ^c^	+12.96 ^b^	+31.48 ^a^	−61.11 ^e^	−24.07 ^d^	−3.70 ^c^
T. Chls.	2.48 ^c^	+8.47 ^b^	+17.74 ^a^	−60.89 ^e^	−22.58 ^d^	−3.63 ^c^
T. Carot.	0.62 ^c^	+11.29 ^b^	+25.81 ^a^	−43.55 ^e^	− 17.74 ^d^	+11.29 ^b^
iEC	0.26 ^c^	+15.38 ^b^	+26.92 ^a^	−53.85 ^e^	−19.23 ^d^	−3.85 ^c^
Fv/Fm	0.82 ^b^	+6.10 ^a^	+8.54 ^a^	−46.34 ^d^	−10.98 ^c^	−2.44 ^b^
Photochemical activity	44.8 ^c^	+11.38 ^b^	+22.10 ^a^	−45.98 ^e^	− 17.86 ^d^	−2.90 ^c^
RWC (%)	79.8 ^b^	+8.90 ^a^	+15.04 ^a^	−40.85 ^d^	−11.78 ^c^	−1.75 ^b^
Proline content	94.2 ^e^	+12.31 ^d^	+34.39 ^c^	+37.79 ^c^	+72.40 ^b^	+91.83 ^a^
T.S. sugars content	12.3 ^e^	+28.46 ^d^	+56.10 ^c^	+72.36 ^c^	+98.37 ^b^	+125.20 ^a^
GB content	122.4 ^f^	+21.41 ^e^	+42.57 ^d^	+57.35 ^c^	+75.49 ^b^	+96.65 ^a^
Choline content	198.2 ^a^	−11.00 ^b^	−21.64 ^b^	−34.61 ^c^	−46.77 ^d^	−58.53 ^e^
K^+^ ion content	2.42 ^c^	+11.57 ^b^	+23.14 ^a^	−57.02 ^e^	−12.40 ^d^	−0.83 ^bc^
Na^+^ ion content	0.32 ^d^	−9.38 ^de^	−21.88 ^e^	+2975.00 ^a^	+1018.75 ^b^	+225.00 ^c^
K^+^/Na^+^ ratio	7.56 ^c^	+23.15 ^b^	+57.67 ^a^	−98.54 ^f^	−92.20 ^e^	−69.44 ^d^
Cl^−^ ion content	0.74 ^d^	−2.70 ^d^	−14.86 ^e^	+1629.73 ^a^	+470.27 ^b^	+108.11 ^c^
O_2_^•^^‒^ level	0.55 ^d^	−12.73 ^e^	−25.45 ^f^	+114.55 ^a^	+36.36 ^b^	+9.09 ^c^
H_2_O_2_ level	1.92 ^d^	−12.50 ^e^	−35.42 ^f^	+198.96 ^a^	+57.29 ^b^	+13.54 ^c^
MDA level	0.18 ^d^	−22.22 ^e^	−38.89 ^f^	+133.33 ^a^	+72.22 ^b^	+22.22 ^c^
IL (%)	6.96 ^c^	−12.07 ^d^	−22.13 ^e^	+250.86 ^a^	+32.47 ^b^	+2.01 ^c^
SI (%)	73.8 ^c^	+8.13 ^b^	+15.45 ^a^	−47.97 ^e^	−18.43 ^d^	−2.03 ^c^
AsA content	3.20 ^e^	+11.88 ^d^	+23.75 ^c^	+27.50 ^c^	+43.13 ^b^	+54.69 ^a^
GSH content	1.31 ^f^	+18.32 ^e^	+34.35 ^d^	+51.15 ^c^	+77.10 ^b^	+100.00 ^a^
α-Tocopherol	46.2 ^e^	+2.60 ^d^	+21.86 ^c^	+26.84 ^c^	+40.26 ^b^	+54.98 ^a^
Phenolic content	41.8 ^e^	+0.24 ^e^	+3.59 ^d^	+24.88 ^c^	+37.32 ^b^	+50.24 ^a^
Capsaicin content	0.84 ^d^	−2.38 ^d^	+1.19 ^d^	+11.90 ^c^	+26.19 ^b^	+45.24 ^a^
Sm content	25.4 ^f^	+12.99 ^e^	+26.77 ^d^	+48.03 ^c^	+70.08 ^b^	+102.36 ^a^
SOD activity	32.2 ^f^	+10.56 ^e^	+21.74 ^d^	+36.65 ^c^	+48.45 ^b^	+62.42 ^a^
CAT activity	4.94 ^f^	+23.89 ^e^	+55.47 ^d^	+94.74 ^c^	+131.58 ^b^	+174.90 ^a^
POD activity	14.6 ^f^	+15.75 ^e^	+28.77 ^d^	+52.05 ^c^	+73.97 ^b^	+104.11 ^a^
APX activity	12.2 ^f^	+13.93 ^e^	+29.51 ^d^	+44.26 ^c^	+67.21 ^b^	+103.28 ^a^
IAA content	15.8 ^b^	+8.86 ^a^	+10.76 ^a^	−41.77 ^d^	−8.23 ^c^	+1.90 ^b^
GAs content	32.1 ^b^	+10.90 ^a^	+15.26 ^a^	−51.09 ^d^	−12.15 ^c^	−0.93 ^b^
Cytokinin content	17.2 ^c^	+15.12 ^b^	+32.56 ^a^	−50.00 ^e^	−19.77 ^d^	−1.74 ^c^
Salicylic acid	148 ^e^	+14.19 ^d^	+29.73 ^c^	+33.78 ^c^	+52.70 ^b^	+72.30 ^a^
ABA content	5.12 ^c^	−36.91 ^d^	−58.20 ^e^	+150.39 ^a^	+21.88 ^b^	+1.17 ^c^

Means (±SE) in each column followed by different letters are significant at the *p* ≤ 0.05 level based on Fisher’s least significant difference (LSD) tests.

## Data Availability

The data presented in this study are available upon request from the corresponding author.

## Supplementary Materials

The following are available online at https://www.mdpi.com/article/10.3390/plants10112316/s1, Table S1: A preliminary study conducted to assess the effect of salinity (5, 10, or 15 dS m^‒1^), BH (Bee-honey at 10, 15, or 20 mL L^−1^), and BH-Sm (Bee-honey at 10 mL L^−1^ + 0.25 mM silymarin) levels on some growth traits, instantaneous carboxylation efficiency (iCE), and chlorophyll content of chili pepper (*Capsicum frutescence* L.) plants.
