# Supplementary material for: Natural Biostimulant Attenuates Salinity Stress Effects in Chili Pepper by Remodeling Antioxidant, Ion, and Phytohormone Balances, and Augments Gene Expression
Source: Plants (Basel). 2021 Oct 27;10(11):2316. doi: 10.3390/plants10112316 (PMC8617650; doi:10.3390/plants10112316)
Supplement: Supplementary file 1 [file plants-10-02316-s001.zip › plants-1397037-supplementary.pdf]

Table S1. A preliminary study conducted to assess the effect of salinity (5, 10, or 15 dS m^{-1}), BH (Bee-honey at 10, 15, or 20 mL L^{-1}), and BH-Sm (Bee-honey at 10 mL L^{-1} + 0.25 mM silymarin) levels on some growth traits, instantaneous carboxylation efficiency (iCE), and chlorophyll content of chili pepper (*Capsicum frutescence* L.) plants

Treatments	Total leaf area $plant^{-1}$ (m^2)	Shoot dry weight $plant^{-1}$ (g)	Root dry weight $plant^{-1}$ (g)	iCE (μmol m^{-2} s^{-1})	Chlorophyll content (mg g^{-1} FW)
Control	329±22a	6.07±0.35a	3.44±0.20a	0.26±0.02a	2.32±0.09a
Salinity(1)	263±14b	4.12±0.21b	2.09±0.12b	0.15±0.01b	1.11±0.04b
**Salinity(2)**	**81±3c**	**1.07±0.08c**	**0.91±0.10c**	**0.06±0.00c**	**0.51±0.02c**
Salinity(3)	**15±0**d	**0.11±0.0**d	**0.09±0.0**d	**0.0±0.0**d	**0.03±0.0**d
Control	329±22c	6.07±0.35c	3.44±0.20c	0.26±0.02c	2.32±0.09c
BH(1)	368±26b	5.09±0.30b	3.15±0.17b	0.29±0.01b	2.48±0.10b
**BH(2)**	**392±32a**	**5.60±0.44a**	**4.02±0.24a**	**0.33±0.02a**	**2.71±0.10a**
BH(3)	401±34a	5.55±0.46a	4.07±0.30a	0.32±0.02a	2.74±0.12a
Control	329±22c	6.07±0.35c	3.44±0.20c	0.26±0.02c	2.32±0.09c
BH-Sm(1)	388±30b	5.38±0.31b	3.10±0.18b	0.30±0.01b	2.54±0.11b
**BH-Sm(2)**	**445±38a**	**6.25±0.52a**	**4.12±0.31a**	**0.34±0.01a**	**2.88±0.13a**
BH-Sm(3)	451±40a	6.27±0.56a	4.16±0.30a	0.34±0.01a	2.89±0.13a
BH-Sm(4)	448±40a	6.27±0.54a	4.18±0.34a	0.35±0.01a	2.87±0.14a

The same letters with mean values±SE in each column indicate non-significant differences, and different letters indicate significant differences according to LSD test ($P \leq 0.05$).

Salinity(1)= 5 dS m^{-1} (3200 ppm NaCl), Salinity(2)= 10 dS m^{-1} (6400 ppm NaCl), Salinity(3)= 15 dS m^{-1} (9600 ppm NaCl), BH(1)= Bee-honey at 10 mL L^{-1}, BH(2)= Bee-honey at 15 mL L^{-1}, BH(3)= Bee-honey at 20 mL L^{-1}, BH-Sm(1)= Bee-honey at 10 mL L^{-1} + 0.25 mM silymarin in the same solution, BH-Sm(2)= Bee-honey at 10 mL L^{-1} + 0.5 mM silymarin in the same solution, BH-Sm(3)= Bee-honey at 15 mL L^{-1} + 0.25 mM silymarin in the same solution, BH-Sm(4)= Bee-honey at 15 mL L^{-1} + 0.5 mM silymarin in the same solution.
